# Miniaturized versus conventional cardiopulmonary bypass in patients undergoing coronary artery bypass surgery: impact on lymphocyte depletion and sternal wound healing

**DOI:** 10.1186/1749-8090-10-S1-A322

**Published:** 2015-12-16

**Authors:** Fabio Capuano, Andrea Lechiancole, Emiliano Angeloni, Massimo Goracci, Roberto Bianchini, Antonino Roscitano, Cosimo Comito, Giovanni Melina, Riccardo Sinatra

**Affiliations:** 1 Department of Cardiac Surgery, University of Rome "La Sapienza", Sant' Andrea Hospital, Rome, 00135, Italy

## Background/Introduction

To reduce deleterious effects of C-CPB novel concepts have been developed based on miniaturized cardiopulmonary bypass (Mini-CPB) with closed circuits, low priming volumes and optimized perfusion system. In CABG surgery, it has previously shown that the use of Mini-CPB can reduce systemic inflammation compared to C-CPB [25] and so attenuate the pathologic effects of C-CPB.

## Aims/Objectives

The aim of this study was to compare miniaturized cardiopulmonary bypass (Mini-CPB) versus conventional cardiopulmonary bypass (C-CPB) in patients undergoing coronary artery bypass grafting (CABG) in term of sternal wound healing and lymphocyte depletion.

## Method

A total of 847 patients undergoing isolated coronary artery bypass grafting (CABG) surgery were studied. Exclusion criteria were: redos, emergencies, CPB time longer than 180 min, antibiotic therapy within two weeks prior to surgery. Finally, 697 consecutive patients who underwent CABG, between January 2012 and September 2014, were studied prospectively. C-CPB was used in 397 (56.9%) patients (Group A) and Mini-CPB was used in 300 (43.1%) (Group B). Patients in the two groups were similar with respect to demographic and preoperative status. To detect lymphocyte depletion, blood was sampled for lymphocyte measurements at three time points: preoperatively (T1), 24 (T2) and 72 h postoperatively (T3) The presence of infections was evaluated according to the ASEPSIS wound scoring system. Antibiotic prophylaxis with cefazolin was performed preoperatively, according to the routine of the institution.

## Results

The study groups had similar EuroSCOREs. A total of 26/697 (3.7 %) patients had sternal wound infection (SWI). Patients from Group A showed a higher incidence of SWI compared to Group B (26/397, 56.5 % vs 0/300, 0% respectively, p = 0.002). In Group A 14/26 (54 %) patients developed deep SWI, and 12/26 (46 %) developed superficial SWI. The Group A but not the Group B showed significant lymphocyte depletion from preoperative during the 1st postoperative day (7.96 ± 4,85 % in Group A vs. 15.4 ± 4.8 % in Group B, p: < 0.0001). Also in 3rd postoperative day, lymphocyte depletion was lesser in Group B (9.83 ± 6.61 % in Group A vs. 13.67 ± 5.41 in Group B, respectively, p < 0.0001). The most frequently cultured isolated were Staphylococcus epidermidis (37%), Staphylococcus aureus (22.2%). 22 (85%) patients were treated by debridement and vacuum therapy and 4 (15%) patients underwent surgical sternal reconstruction.

## Discussion/Conclusion

This study shows that Mini-CPB for patients undergoing isolated CABG is associated with a reduced risk of SWI occurrence. This may be related to the lesser inflammatory response of Mini-CPB compared to C-CPB and to the lesser lymphocyte depletion. Further studies are needed to confirm these findings.

